# Estimating the use of biological samples in Finnish biobanks and hospital collections

**DOI:** 10.1038/s41431-025-01906-w

**Published:** 2025-07-05

**Authors:** Aaro Tupasela, Tom Southerington, Johanna Mäkelä, Lila Kallio, Merja Perälä, Veli-Matti Kosma, Arto Mannermaa, Tiina Jokela, Kimmo Pitkänen, Mika Kontro, Tiina Vesterinen, Eero Punkka, Theresa Knopp, Minna Ruddock, Raisa Serpi, Anne-Mari Moilanen, Leena Viiri, Sanna Siltanen, Enni Makkonen, Päivi Ingalsuo

**Affiliations:** 1 Faculty of Social Science, University of Helsinki, Helsinki, Finland; 2 Finnish Biobank Coopereative (FINBB), Turku, Finland; 3 University of Turku, Turku, Finland; 4 Auria Biobank, University of Turku and Turku University Hospital, Turku, Finland; 5 Biobank of Eastern Finland, Wellbeing Services County of North Savo, North Savo, Finland; 6 Central Finland Biobank, Wellbeing Services County of Central Finland, Jyväskylä, Finland; 7 Blood Service Biobank, Finnish Red Cross Blood Service, Vantaa, Finland; 8 The Finnish Hematology Registry and Clinical Biobank, Helsinki, Finland; 9 Helsinki Biobank, Helsinki, Finland; 10 Arctic Biobank, Faculty of Medicine, University of Oulu, Oulu, Finland; 11 Biobank Borealis of Northern Finland, Wellbeing services county of North Ostrobothnia, Oulu, Finland; 12 Finnish Clinical Biobank Tampere, Tampere University Hospital, Wellbeing Services County of Pirkanmaa, Tampere, Finland; 13 THL Biobank, Helsinki, Finland

**Keywords:** Population genetics, Social sciences, Genetics research

## Abstract

Finland has steadily developed its biobanking infrastructures since the early 2010s. This article presents a systematic overview of the number of biological samples that have been provided for research since 2013 when the Finnish Biobank Act came into force. Using data from individual biobanks and from permits issued by Fimea (formerly Valvira), we present the most up-to-date and complete account of sample use at a national level. Between 2014 and 2023 a total of 1,474,881 samples were provided through 998 sample requests. A better understanding of the use of biological samples at the national level can help highlight the important impact biobanking has as an infrastructure for supporting research. We argue that developing standards can help in developing national biobanking strategies and identify areas where biobanking can be further developed. We also conclude that the ability to combine tissue samples and health data flexibly and efficiently is essential and needs to be secured also within the context of the European Health Data Space (EHDS).

## Introduction

During the past two decades, biobanking has become increasingly professional where the collection, storage and distribution of samples has become more systematic, of high quality, and designed increasingly to meet the needs of the research community [1–3]. In some of the Nordic countries, such as Finland and Denmark, biobanking activities are increasingly being coordinated to facilitate easy identification of and access to samples at a national scale by developing portals or gateways to help identify samples and sample or donor related data. Internationally, organization such as BBMRI-ERIC have set up sample catalogs to help researchers identify samples stored in different biobanks [4]. In Finland, the Finnish Biobank Cooperative – FINBB offers centralized access to public Finnish biobank collections (www.fingenious.fi.). The goal of these efforts has been to facilitate a more efficient use of existing resources and thus increase the impact of existing resources.

Despite the increased coordination around biobanking, the assessment of impact has been a major challenge within the biobanking community for quite some time [5]. Metrics regarding publications, for example, suggest above average increase compared to other fields [6]. Numerous studies and single collections or biobanks have reported and sought to estimate levels of sample usage in countries such as Denmark [7, 8], and the UK [9]. Other studies have sought to calculate the scientific impact of data and sample use related to a single collection [10] or surrounding a specific research area such as oncology [11]. These studies, however, reflect estimates of human biological sample use involving a single collection or a biobank. This has also given rise to discussions regarding the appropriate way of estimating sample use [12]. Should biobanks be required to systematically report on sample use and other meta-data regarding their operations or does this pose too much of a burden? Despite being a critical infrastructure within the biomedical and personalized medicine discovery process, there is little data at the national level on sample use for biomedical research.

Some of this is due to the fragmented and local nature of sample collections, as well as the lack of standardization on how sample use estimates could be done. In many cases, sample collection and distribution are handled by different types of intermediaries, such as pathology departments [13]. In addition, despite advances in standardization among biobanks [14] there is still a lack of data collection standards on metrics which would allow for meaningful comparisons between sample collections [15], not to mention countries. Furthermore, it is unclear which metrics are meaningful for measuring biobank impact in the first place [11].

The aim of this study was to collect data on the use of biological samples, which fall under the Biobank Act and the Tissue Act in Finland. This article presents the findings on the use of biological samples, such as tissue, cells, and blood in addition to their derivatives at the national level from 2013 to the end of 2023. We present data collected from 10 biobanks and tissue use permits granted by Fimea (formerly Valvira). Although these figures, do not represent all the biological samples that are used for research in Finland, to our understanding, this is the first study of its kind to present data on sample usage at the national level and at such a level of detail. The survey of sample use at the national level in Finland is important in understanding both the impact that biobanking has on biomedical research, but also helps identify important policy areas where biobanking can be further supported.

## The Finnish context

The first approved and registered biobanks under the current Biobank Act (see below) were the Auria biobank, the The Finnish Institute for Health and Welfare (THL) biobank and the The Finnish Hematology Registry and Clinical Biobank (FHRB Biobank) in 2014. These were followed by the Borealis, Helsinki, Tampere, Eastern Finland, and Central Finland biobanks in 2015. In 2017 The Finnish Red Cross Blood Service, Blood Service Biobank (FRCBS, BSB) and the private Terveystalo biobanks were established. The Arctic Biobank – University of Oulu was established in 2020.

After being established these biobanks began to collect samples using a broad biobank consent model. Approximately 10 million so-called legacy samples, have been transferred from e.g. hospital archives to the biobanks when they were established. These samples include old diagnostic or research samples that were transferred to biobanks in accordance with the Biobank Act [16]. Those diagnostic and therapeutic samples which have not been transferred to the biobanks can still be accessed in accordance with the Tissue Act.

An important contributor to the use of biobank samples in Finland has been the FinnGen project (https://www.finngen.fi/en), which begun in 2017 and has to date successfully genotyped over 500,000 samples provided by the different biobanks in Finland. The genome data produced from the project and returned to biobanks has become an important source of subsequent data requests to biobanks. The returned data serves also as an important driver for international collaborations.

In 2017, the six largest Finnish regional healthcare authorities and six leading Finnish universities founded FINBB to act as a joint service provider to Finnish biobanks. The THL, a governmental agency, joined as a member and owner in 2019. The FINBB biobank network comprises the eight biobanks of its members, and it collaborates closely with the three other registered Finnish biobanks. In 2020, the Finnish Ministry of Education appointed FINBB as the coordinating national node for BBMRI-ERIC, a European research infrastructure for biobanking.

FINBB aims at harmonizing Finnish biobank operations and improving access to samples, data and services for health-related research. Its core activity is providing researchers centralized access to the collections and services of the Finnish biobanks and health care providers. This it achieves through its continuously evolving Fingenious® digital services (www.fingenious.fi), which include for example finding and obtaining suitable biobank samples and EHR and other associated health data, as well as patient finding for clinical trial recruitment.

## Biobank legislation in Finland

The use of human biological samples in biomedical research is not new, however, data collection on the use of samples in Finland began only in 2001 with the Tissue Act, which authorized the National Authority for Medicolegal Affairs (TEO) to grant permits for the use of human biological samples. In 2009 TEO was re-named as Valvira and in 2020 the permit authority for tissue use was moved to the Finnish Medicines Agency (Fimea).

During the studied period, Finland also enacted the Biobank Act that came into force on 1 September 2013. The main driving force behind the Act was to develop a comprehensive legislation which would address the concerns of the biomedical research community regarding what was a narrow interpretation of informed consent regarding older samples. In this interpretation the scope of informed consent was considered too narrow for the secondary use of samples and data [17]. Through the Biobank Act, any legal entity that meets the requirements can establish a biobank regardless of whether they are a public or private entity. Fimea approves biobanks’ registrations and oversee their functions. The Biobank Act introduced several new instruments which allowed for the transfer of legacy collections to biobanks without gaining re-consent, as well as introduced amendments to the existing Tissue Act. In addition, the Biobank Act allowed biobanks to collect samples using broad biobank consent for future research, as well as an opt-out mechanism through which donors can exclude existing samples [18]. At the same time, it created a legal framework for the establishment of institutional biobanks, which would be charged with the management of samples and related data. The combination of samples and related data, and the possibility to obtain them together from the biobanks was especially important, enabling the selection of research samples with the aid of clinical and other relevant data, and obtaining research samples and relevant data more easily [19].

The scope of the Tissue Act and the Biobank Act are only partly overlapping. The Tissues Act covers secondary uses of tissue from diagnostic and therapeutic use and medical autopsies, while the Biobank Act covers also reusing research samples, and using samples collected particularly for the biobank for future research purposes. The Biobank Act increased the number of samples available for research, as well as of associated sample and health related data. Most biobank samples used in research during the studied period were samples that could not have been accessed through the Tissues Act, because they were not originally collected for health care purposes. The Biobank Act has therefore played an important role in developing biological sample use in Finland, but the Tissue Act also continues to serve researchers who seek samples, which have not been transferred to biobanks.

## Materials and methods

Since 2013 when Finland introduced the world’s first legislation regarding the institutionalization of biobanks, researchers have been able to request the use of human-derived tissue samples from two main sources. The first is the biobanks themselves and the second is from the National Supervisory Authority for Welfare and Health (Valvira), and the Finnish Medicines Agency (Fimea). Our findings on estimating the use of human samples in Finland are based on data collected from these two main sources.

The first data has been collected from official permits that have been given for the secondary use of human tissue (Act on the Medical Use of Human Organs, Tissues and Cells 101/2001, as amended, “Tissue Act”101/2001 and 202/2019). This data is available since 2001 [20]. The granting authority for these permits has changed three times since 2001 (TEO 2001–2008; Valvira 2009–2019; Fimea 2020–). The data was collected by going through each application and counting the number of samples that were being requested for re-use. These figures should be seen as estimates since the applications do not have a standard according to which samples are counted. In some applications the permit request was to re-use samples from a single patient, whereas in other applications the request was for a specific number of samples. The original data set collected included how many projects were granted a permit to re-use samples, how many samples/patients this included, the general area of research (cancer, diabetes…) and whether samples were to be shared internationally. Many of the samples that are applied for through this channel include FFPE samples, as well as slides in the hospital collections. These collections do also contain DNA, serum, and punch cards to name a few. Unfortunately, there is no accurate estimate of how many samples are available in hospital collections, but some estimates suggest that there are millions [21].

The second source of data have been provided by ten Finnish biobanks (four national biobanks and six regional hospital biobanks, one private health care provider biobank has been in operation, but its sample numbers would not significantly affect the analyses). The data has been compiled with the help of the Finnish Biobank Cooperative (FINBB, https://finbb.fi/en/) using a standardized format developed when data was collected from permits for the secondary use of human tissue. The provision of this data by biobanks was a challenge since there is no standardized format which biobanks use to collect meta-data on their sample use and not all biobanks were able to provide all the requested information, such as whether there was international collaboration or not. We have, therefore chosen to provide a baseline of information which include data on how many research material requests have received samples and how many samples have been supplied by the biobanks.

The sample collections included DNA, serum, plasma, buffy coat, whole blood, PBMCs, punch cards, tissue slides and FFPE tissue blocks. Biobanks increasingly maintain and share data from previous studies since researchers are expected to return the analysis results to the biobanks, and samples are typically also accompanied by relevant data from health registers. This article, however, focuses specifically on physical samples; a study on data requests and permits is the topic of a subsequent study. Furthermore, our study does not include tissue or organs used for transplant or therapeutic purposes, nor does it include tissue or cells collected from reproductive therapies. There are slight discrepancies regarding samples from deceased individuals.

Biobanks are allowed to provide these samples if they are part of a collection that has been transferred to the biobank or the deceased has provided an informed biobank consent while alive.

Regarding samples under the Tissue Act, we have not included samples collected after 2017 from deceased individuals. After 2017, these permits have gone through statutory regional Medical Ethics Committees (EC). The regional EC’s do not collect any statistical data on the number of samples from deceased individuals that are being re-used. Furthermore, we do not have any data on the samples that pharmaceutical companies collect and use for their own clinical research.

## Results – biological sample use in Finland

The Biobanking Act came into force in 2013, and the first biobanks were established a year later. Between 2014 and 2023 a total of 998 permits were given for the secondary use of human samples from biobanks and Fimea (Formerly Valvira) (see Table 1). From the total number, biobank permits made up 847 permits, while Fimea accounted for 151 permits in total.

**Table 1:** Number of permits granted for sample use 2014–2023.

Year	FIMEA	Arctic	Auria	Borealis	Helsinki biobank	Biobank of Eastern Finland	Central Finland biobank	Finnish clinical biobank Tampere	THL biobank	Blood service biobank	FHRB biobank	
2014	22		6									
2015	20		37								2	
2016	21		26		1				8		6	
2017	16		35	4	11	2			4	1	4	
2018	11		33	7	19	2	1	2	7	0	4	
2019	16		40	27	22	4	5	15	4	1	4	
2020	10		27	28	35	11	5	18	2	4	7	
2021	8	1	26	20	33	12	3	16	4	4	4	
2022	14	1	38	16	35	12	2	12	4	1	3	
2023	13	1	23	32	39	6	7	5	2	1	3	Total
Total	151	3	291	134	195	49	23	68	35	12	37	998

Of all the permits that were issued, a total of 1,496,453 samples were provided for research (excluding permits for bodies used for teaching purposes) (see Table 2). From the total number, Finnish biobanks provided a total of 685,067 samples, while Fimea provided permits to a total of 811,386 samples. For all biobanks the average sample size per permit was 808 samples over all the years, while for Fimea this was 5373 samples per project. For the purposes of comparison, before the establishment of biobanks in Finland, from 2001 to 2011 a total of 273 projects were granted the re-use of 646,911 samples by TEO and Valvira for research.

**Table 2:** Number of biological samples provided for research 2014–2023.

Year	FIMEA	Arctic	Auria	Borealis	Helsinki biobank	Biobank of Eastern Finland	Central Finland biobank	Finnish clinical biobank Tampere	THL biobank	Blood service biobank	FHRB biobank	
2013												
2014	11,087											
2015	11,553		246								430	
2016	14,063		2636		151				7440		284	
2017	28,652		5217	118	896	30			44,180	1465	775	
2018	28,713		21,836	3808	27,867	2060	7	2062	34,000	16,827	496	
2019	32,158		18,709	12,615	38,848	5938	5575	6263	10,981	23,155	152	
2020	20,289		13,528	28,481	31,994	4034	3648	18,749	14,802	19,437	2261	
2021	4635	6051	10,639	16,317	20,942	11,773	2601	15,247	8149	10,677	184	
2022	49,480	234	14,982	8509	24,883	9558	2333	4148	15,572	12,243	869	
2023	610,756	203	3930	7547	15,238	1728	1111	262	3889	1497	178	Total
Total	811,386	6488	91,723	77,395	160,819	35,121	15,275	46,731	139,013	85,301	5629	1,474,881

During this period and among both Fimea and biobank permits, the smallest sample request was for two samples, while the largest request was for 600,000 samples. Most of the applications for permit were for samples pertaining to cancer. In the Auria Biobank, for example, slightly over 60% of research projects relate to cancer research followed by, blood disorders (4%), other diseases (4%), neurological diseases (3%), and respiratory diseases (3%) [22]. Similarly at the Helsinki Biobank, research on cancer account for about 50% of all studies, followed by cardiovascular diseases (15%), and neurological diseases (10%) [23]. For the studies that have requested samples through Fimea roughly 60% represent studies on cancers.

Over the years, the number of permits granted by Fimea have fluctuated somewhat, but the demand for samples which are not located in biobanks has not decreased despite the transfer of a significant number of legacy samples to biobanks, as well as the growing importance of biobanks as providers of samples and related data. Both within the biobank requests and the Fimea requests two factors have attributed to requests amounting to a total of 1,1 million samples. Within the biobank requests, the FinnGen project has requested a total of over 500,000 samples and from Fimea a single project requested 600,000 samples. The effect of these two request sources has been significant regarding the total number of requests. As mentioned, most of the biobank samples were samples that were never under the Tissue Act, so the overall number of tissue samples for research grew with the biobanks.

**Fig. 1:**
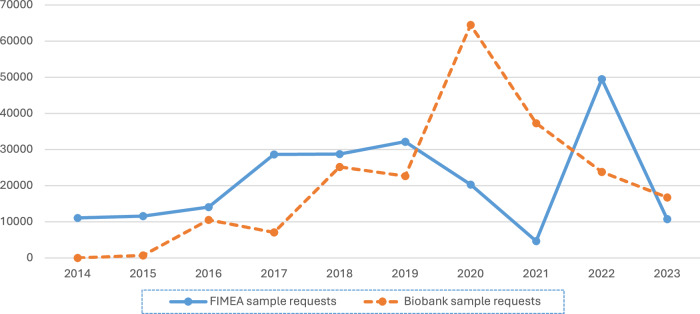
Number of biological samples provided for research 2014–2023 by Fimea and biobanks (Adjusted for large projects).

For biobank samples the number of samples provided for research saw a steady increase until 2020 when a total of 147,561 samples were provided for research. Since then, there has been a decline in the number of samples provided for research. Two factors have contributed to this decrease; an increasing number of the biobank requests can be met through the provision of data that has been returned to the biobanks from previous studies, and the decrease in requests from Finngen, which contributed to a total of over 500,000 sample requests over several years. In Fig. 1, we have plotted the number of samples provided by all biobanks and Fimea without the effect of the Finngen requests (500,000 samples) and one large request from Fimea (600,000 sample).

Between the biobanks themselves, there are differences in the number of projects that have been granted permits, as well as the number of samples which have been requested and used. It should be noted, however, that these figures can be misleading since many of the biobanks increasingly provide data that has already been extracted from samples. Furthermore, projects and sample requests and deliveries are often undertaken over several years during which the samples are used, so yearly statistics should be understood only as being indicative of the impact and significance that biobanking has on research.

## Discussion

Biobanks and different sample collections play an important role in facilitating biomedical research. There is, however, a significant lack of standardized, comprehensive, and comparable data regarding the amounts of tissue samples that are being used for biomedical research. This problem is in part due to the fragmented nature of biobanking. Data on sample usage can serve as an important metric for the assessment of impact and help support policymaking and funding decisions. The establishment of common metrics on sample use and related meta-data should be a priority among the biobanking community. Organizations, such as BBMRI, could play an increasingly important role in facilitating such standardization in Europe. Important metrics that should be included are how many samples are used, what types of samples are being used, what types of research fields are samples being used for, as well as are samples being requested by public, private or joint research projects.

It should be noted, however, that looking at tissue use numbers alone can be misleading since biobanks are increasingly becoming important repositories of different kind of health-related or returned data that can be requested for further biomedical and biobank research. Another finding of this study relates to the metrics used to evaluate the impact of biobanks. The FinnGen project, for example, lists over 1000 articles that have been published using the data that it has produced. It is unclear, however, how biobanks should calculate the impact of the samples they have provided since the samples come from all the major biobanks in Finland. The study also indicated the biobanks and tissue collections in hospitals serve a broad range of uses where sample requests may range from a few samples to tens of thousands of samples and related data. The role of samples in care and treatment is still not properly understood and requires further research. Therefore, the significance of biobanking services cannot be understated, but the measurement of impact remains difficult to calculate in a meaningful way.

Collecting and presenting systematic data of sample use is also an important element of biobank transparency and helps to strengthen the social responsibility exercised by biobanks as they continue to operate and function as important parts of the medical field. Transparency also helps to provide important justifications for public support of biobanking activities. The data from Finland suggests that it is becoming increasingly important to raise awareness of national resources – both physical samples and related health and registry data - given their importance in relation to the EHDS. Finally, collecting comprehensive data on biobanking activities also helps to identify future policy needs and trends which may require further public support, as well as possible adjustments to regulations and practice. One area that may require more investment is to provide sufficient funding for biobank support services, including the collection of metadata and data curation.

## Data Availability

The datasets generated and/or analyzed during the current study are available from the corresponding author on reasonable request.
